# *Toxoplasma gondii* Cyclic AMP-Dependent Protein Kinase Subunit 3 Is Involved in the Switch from Tachyzoite to Bradyzoite Development

**DOI:** 10.1128/mBio.00755-16

**Published:** 2016-05-31

**Authors:** Tatsuki Sugi, Yan Fen Ma, Tadakimi Tomita, Fumi Murakoshi, Michael S. Eaton, Rama Yakubu, Bing Han, Vincent Tu, Kentaro Kato, Shin-Ichiro Kawazu, Nishith Gupta, Elena S. Suvorova, Michael W. White, Kami Kim, Louis M. Weiss

**Affiliations:** a Department of Pathology, Albert Einstein College of Medicine, Bronx, New York, USA; b National Research Center for Protozoan Diseases, Obihiro University of Agriculture and Veterinary Medicine, Obihiro, Hokkaido, Japan; c Department of Microbiology and Immunology, Albert Einstein College of Medicine, Bronx, New York, USA; d Department of Molecular Parasitology, Humboldt University, Berlin, Germany; e Departments of Molecular Medicine and Global Health, University of South Florida, Tampa, Florida, USA; f Department of Medicine, Albert Einstein College of Medicine, Bronx, New York, USA

## Abstract

*Toxoplasma gondii* is an obligate intracellular apicomplexan parasite that infects warm-blooded vertebrates, including humans. Asexual reproduction in *T. gondii* allows it to switch between the rapidly replicating tachyzoite and quiescent bradyzoite life cycle stages. A transient cyclic AMP (cAMP) pulse promotes bradyzoite differentiation, whereas a prolonged elevation of cAMP inhibits this process. We investigated the mechanism(s) by which differential modulation of cAMP exerts a bidirectional effect on parasite differentiation. There are three protein kinase A (PKA) catalytic subunits (*Tg*PKAc1 to -3) expressed in *T. gondii*. Unlike *Tg*PKAc1 and *Tg*PKAc2, which are conserved in the phylum Apicomplexa, *Tg*PKAc3 appears evolutionarily divergent and specific to coccidian parasites. *Tg*PKAc1 and *Tg*PKAc2 are distributed in the cytomembranes, whereas *Tg*PKAc3 resides in the cytosol. *Tg*PKAc3 was genetically ablated in a type II cyst-forming strain of *T. gondii* (Pru*Δku80Δhxgprt*) and in a type I strain (RH*Δku80Δhxgprt*), which typically does not form cysts. The Δ*pkac3* mutant exhibited slower growth than the parental and complemented strains, which correlated with a higher basal rate of tachyzoite-to-bradyzoite differentiation. 3-Isobutyl-1-methylxanthine (IBMX) treatment, which elevates cAMP levels, maintained wild-type parasites as tachyzoites under bradyzoite induction culture conditions (pH 8.2/low CO_2_), whereas the Δ*pkac3* mutant failed to respond to the treatment. This suggests that *Tg*PKAc3 is the factor responsible for the cAMP-dependent tachyzoite maintenance. In addition, the Δ*pkac3* mutant had a defect in the production of brain cysts *in vivo*, suggesting that a substrate of *Tg*PKAc3 is probably involved in the persistence of this parasite in the intermediate host animals.

## IMPORTANCE

*Toxoplasma gondii* is one of the most prevalent eukaryotic parasites in mammals, including humans. Parasites can switch from rapidly replicating tachyzoites responsible for acute infection to slowly replicating bradyzoites that persist as a latent infection. Previous studies have demonstrated that *T. gondii* cAMP signaling can induce or suppress bradyzoite differentiation, depending on the strength and duration of cAMP signal. Here, we report that *Tg*PKAc3 is responsible for cAMP-dependent tachyzoite maintenance while suppressing differentiation into bradyzoites, revealing one mechanism underlying how this parasite transduces cAMP signals during differentiation.

## INTRODUCTION

*Toxoplasma gondii*, a protozoan pathogen that is a member of the phylum Apicomplexa, is found throughout the world with an estimated 30% seroprevalence in humans (1). This parasite can differentiate between rapidly replicating tachyzoites that cause acute infection and slowly growing bradyzoites found in the tissue cysts (2). Consumption of undercooked meats containing tissue cysts is a significant risk factor for transmission of *T. gondii* (3). There is also a sexual stage, i.e., the oocyst, which develops in cats and that can also transmit infection when it is ingested in contaminated water or food. Primary infection with this parasite during pregnancy can cause congenital infection resulting in spontaneous abortion, stillbirth, or fetopathy (4). Tissue cysts containing bradyzoites persist in the host, causing chronic infection. This latent infection can reactivate, with bradyzoites becoming tachyzoites, leading to encephalitis or other diseases, when the immune system is compromised due to HIV infection, immunosuppressive medications, or other factors (4). A better understanding of the molecular mechanisms of parasite differentiation is needed to elucidate the pathogenesis of this infection and for the development of new therapeutic approaches to eliminate latency.

Previous reports have shown that physicochemical stress can induce bradyzoite differentiation in tissue culture (5). A shift to high pH (i.e., pH 8.2), which is widely used to induce bradyzoites, causes a short-term upregulation of cyclic AMP (cAMP) levels in parasitized cultures (6). An optogenetically induced short-term elevation of cAMP within the parasite has been demonstrated to promote bradyzoite formation (7). While a transient cAMP pulse induces bradyzoites, a prolonged induction of cAMP results in inhibition of differentiation (6, 7), suggesting the presence of bidirectional cAMP-induced regulatory mechanisms that may be differentially responsive to the duration or kinetics of cAMP availability.

In eukaryotic cells, cAMP binds to cAMP-dependent protein kinase A (PKA) regulatory subunits (PKArs), leading to the activation of PKA catalytic subunits (PKAcs) (8). In spite of the similarity among PKAc isoforms in an organism, they are often involved in regulating distinct pathways and responses. For example, the three PKAc isoforms of *Saccharomyces cerevisiae* work distinctly by phosphorylating specific transcription factors during nutrition starvation (9) and in response to various carbon sources (10). Previous work using H89, a small-molecule inhibitor for all of the PKAc isoforms, demonstrated that PKAcs in *T. gondii* play roles in regulating the rate of cell division (11) and bradyzoite differentiation (6, 12). In *Plasmodium falciparum*, *Pf*PKA has been reported to regulate invasion (13), and *Toxoplasma* invasion has been reported to be affected by PKA signal ablation (7). The PKAc isoforms responsible for these biological functions have not been identified. Furthermore, it remains unclear whether the same PKAc isoform transduces the signal for these distinct biological functions or if different isoforms regulate these biological functions.

To better understand the various functions of the *T. gondii* PKAs, we first identified the PKA catalytic subunits in the *Toxoplasma* genome and then sought to identify catalytic subunit-specific functions in this pathogen.

## RESULTS

### The *Toxoplasma* genome encodes three putative PKA catalytic subunits.

**FIG 1:**
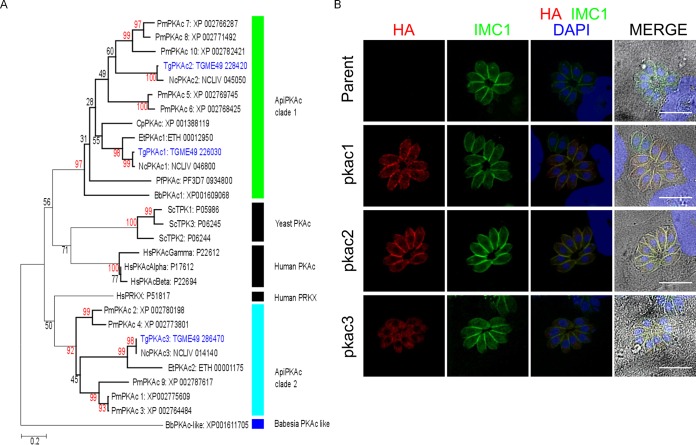
*Tg*PKAc3 is distinct from other PKA catalytic subunits in phylogenetic analysis and localization manner. (A) The phylogenetic tree was calculated with the maximum likelihood method based on the Le-Gascuel 2008 model (28). The tree is drawn to scale, with branch lengths measured in the number of substitutions per site (*Tg*, *Toxoplasma gondii*; *Pf*, *Plasmodium falciparum*; *Bb*, *Babesia bovis*; *Nc*, *Neospora caninum*; *Pm*, *Perkinsus marinus*; *Cp*, *Cryptosporidium parvum*; *Et*, *Eimeria tenella*; *Sc*, *Saccharomyces cerevisiae*; *Hs*, *Homo sapiens*). Bootstrap confidence values above 80% are shown in red. (B) RH*Δku80Δhxgprt* was transfected with C-terminally HA-tagged *Tg*PKAc1, -2, and -3 expression constructs driven by the *GRA1* promoter, inoculated into host cells, and incubated for 24 h. Fixed parasites were stained with anti-HA rat MAb 3F10 and anti-IMC1 rabbit antibody followed by detection with Alexa 594-conjugated anti-rat IgG goat secondary antibody or Alexa 488-conjugated anti-rabbit IgG goat secondary antibody. Nuclei were stained with DAPI. Bars, 10 µm.

Bioinformatic searches identified three distinct PKAc subunits in the *T. gondii* genome (http://www.ToxoDB.org), *Tg*PKAc1 (TGME49_226030), *Tg*PKAc2 (TGME49_228420), and *Tg*PKAc3 (TGME49_286470). Phylogenetic analysis was performed using the PKAc genes from *T. gondii*, *Plasmodium falciparum*, *Babesia bovis*, *Eimeria tenella*, *Neospora caninum*, *Perkinsus marinus*, *Saccharomyces cerevisiae*, and human. This analysis demonstrated that *Tg*PKAc1 and *Tg*PKAc2 are in the same clade as other apicomplexan PKAc genes (ApiPKAc clade 1 [Fig. 1A]). *Tg*PKAc3 and its orthologs from *Neospora* and *Eimeria* are divergent from ApiPKAc clade 1. Transcriptomic data for parasites undergoing sexual development in cat intestine (14) showed high expression of *Tg*PKAc2 during the sexual developmental life cycle of *T. gondii* in cats. *Tg*PKAc1 and *Tg*PKAc3 were expressed during asexual development (i.e., tachyzoites and bradyzoites) (http://www.ToxoDB.org; bradyzoite differentiation, multiple 6-h time points and extended time series from the David Roos Laboratory).

**FIG 2:**
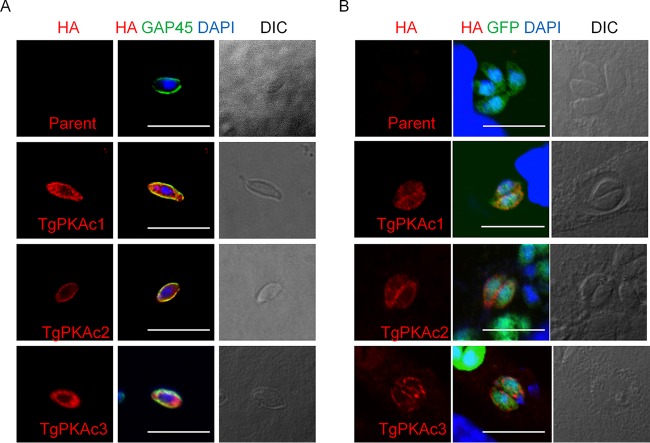
Localizations of *Tg*PKAcs in extracellular and intracellular parasites. Localizations of *Tg*PKAcs-3HA fusion proteins under the extracellular condition (A) and the bradyzoite condition (B) are shown. (A) *Tg*PKAcs-HA expression plasmids were transfected to RH*Δku80Δhxgprt*, and the host monolayer was infected with parasites. After a 24-h incubation, parasites were harvested, purified from host cells, and incubated in the culture medium for 30 min at 37°C on the glass coverslips. Attached parasites were fixed and stained with anti-HA and IMC marker GAP45. (B) Pru*Δku80Δhxgprt* parasites were transfected with *Tg*PKAc-HA expression plasmids and inoculated into the host monolayer. After a 2-h invasion, infected host cells were washed and cultured in pH 8.2 medium under a CO_2_-depletion condition for 48 h. Fixed infected host cells were stained with anti-HA antibody and anti-GFP antibody. Bars, 10 µm. DIC, differential interference contrast.

Next, we checked the localization of each PKA catalytic subunit by expressing hemagglutinin (HA)-tagged PKAc proteins. *Tg*PKAc1-HA and *Tg*PKAc2-HA were primarily detected at the parasite periphery and less prominently in the cytosol and colocalized with inner membrane complex (IMC) marker IMC1, whereas *Tg*PKAc3-HA localized primarily to the parasite cytosol (Fig. 1B). PKA activation is usually regulated by binding of the regulatory subunit of PKA (PKAr) to PKAc. *Tg*PKAr1 localized mainly to the parasite periphery, with increased labeling in the apical end (see Fig. S1 in the supplemental material). When parasites were liberated from host cells, extracellular parasites showed localization patterns similar to those seen with intracellular parasites, i.e., *Tg*PKAc1 and -2 were detected at the periphery of parasites and *Tg*PKAc3 was detected primarily in parasite cytosol (Fig. 2A). When parasites were incubated under bradyzoite culture conditions (pH 8.2, low CO_2_), *Tg*PKAc3 was seen at the periphery of parasites in a punctate pattern (Fig. 2B).

**FIG 3:**
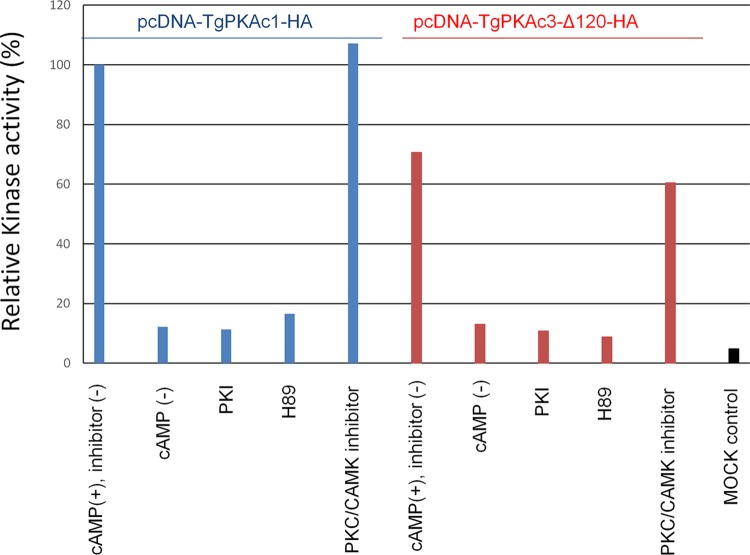
*In vitro* kinase assay of PKAc1 and PKAc3 kinase domain. pcDNA3 mammalian expression plasmid containing coding sequence of HA-tagged PKAc1 and a kinase domain of PKAc3 (PKAc3-Δ120) was transfected to 293T cells, incubated for 48 h, and lysed to purify the recombinant proteins by anti-HA tag immunoprecipitation. The immunoprecipitated *Tg*PKAc1-HA or *Tg*PKAc3-Δ120-HA complex was used for an *in vitro* kinase assay in the presence of 1.66 µM cAMP, 1 µM PKA inhibitor peptide PKI, PKC-CAMK inhibitor mixture (3.3 µM PKC inhibitor peptide and 0.33 µM R24571), or 10 µM H89 or in the absence of cAMP according to the manufacturer’s protocol for the PKA assay kit (Merck Millipore). Incorporation of radioactive ^32^P into the phosphorylated PKA substrate peptide Kemptide was measured with a scintillation counter. Relative PKA kinase activity was normalized with the average value from the PKAc1-HA reaction in the presence of cAMP without any inhibitors. As a control, mock-transfected 293T cell lysate was used for immunoprecipitation, and the kinase assay was performed (rightmost bar). Average values from a representative of two independent experiments are shown.

HA-tagged *Tg*PKAc1 and a kinase domain of *Tg*PKAc3 (*Tg*PKAc3-Δ120Nterm) were expressed in mammalian (293T) cells to examine whether the predicted PKA catalytic domains had kinase activity. Purified *Tg*PKAc1-HA and *Tg*PKAc3-HA proteins bound to anti-HA columns were used for an *in vitro* kinase assay. Both *Tg*PKAc1 and *Tg*PKAc3-Δ120Nterm demonstrated cAMP-dependent kinase activity [Fig. 3, lanes “cAMP(+), inhibitor(-)” and lanes “cAMP(-)”]. The kinase activities of both *Tg*PKAc1 and *Tg*PKAc3-Δ120Nterm were inhibited by the PKA-specific inhibitors H89 and protein kinase inhibitor peptide (PKI), but not by a mixture of protein kinase C (PKC)/Ca^2+^/calmodulin-dependent protein kinase (CAMK) inhibitors (Fig. 3).

Overall, these results demonstrate the presence of at least three PKAc subunits in *T. gondii*, of which *Tg*PKAc3 appears to be distinct from the other two isoforms, based on its phylogenetic divergence and subcellular localization.

### Generation of the *Tg*PKAc3 mutant and complementation strains in the cyst-forming type II *T. gondii* strain Pru*Δku80Δhxgprt*.

**FIG 4:**
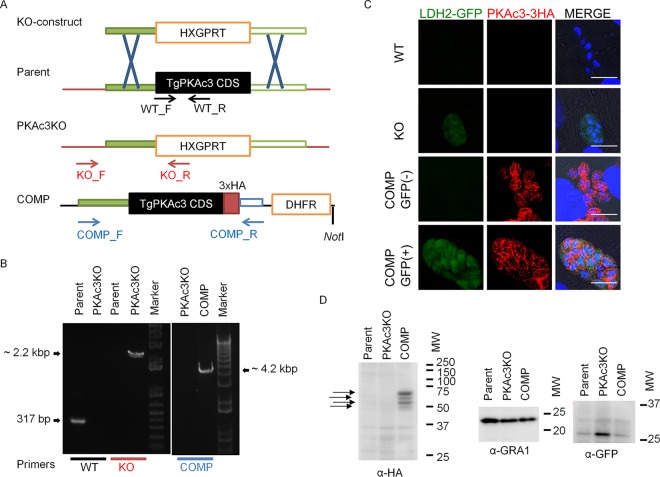
Knockout of *Tg*PKAc3 and its complementation. (A) Schematic depiction of the manipulated *Tg*PKAc3 locus. The knockout construct contains HXGPRT-expressing cassette bound with 5′ untranslated region and 3′ untranslated region of *Tg*PKAc3 (green filled and open boxes, respectively). The genomic locus of *Tg*PKAc3 was replaced with HXGPRT by double homologous recombination and produced PKAc3KO. The complementation construct contains 2.2-kbp 5′ untranslated region and genomic DNA sequence spanning *Tg*PKAc3 coding sequence (CDS) just before the stop codon followed by the 3HA tag (red box) and terminator from HXGPRT (blue open box). The drug selectable marker is shown in the orange open box. The NotI site just after the DHFR selectable marker is shown. Detection primers to check the transgenic parasite clones are shown by arrows. (B) Genomic DNA from parental parasite Pru*Δku80Δhxgprt* (Parent), knockout clone Pru*Δku80Δpkac3* (PKAc3KO), and complemented clone Pru*Δku80Δpkac3*::PKAc3-3HA (COMP) was used for PCR amplification using the primer set described at bottom. (C) Forty-eight hours after parasite inoculation, infected host cells were stained with anti-HA antibody (red). GFP signals driven by LDH2 were enhanced with staining with anti-GFP antibody (green), and nuclei were stained with DAPI (blue). Bars, 10 µm. (D) 3HA-tagged *Tg*PKAc3 was detected from the protein lysate from parental parasite Pru*Δku80Δhxgprt* (Parent), knockout clone Pru*Δku80Δpkac3* (PKAc3KO), and complemented clone Pru*Δku80Δpkac3*::PKAc3-3HA (COMP). Protein lysate from 10^6^ parasites/lane was loaded and detected with anti-HA (left panel), anti-GRA1 (center panel), and anti-GFP (right panel) antibodies. Arrows show the four distinct bands detected in the *Tg*PKAc3-3HA complemented parasite. WT, wild type; KO, knockout; MW, molecular weight in thousands.

To explore the physiological importance of *Tg*PKAc3 for asexual reproduction and differentiation in *T. gondii*, we generated a *Tg*PKAc3 null mutant (Fig. 4A) in the cyst-forming type II strain Pru*Δku80Δhxgprt*, which expresses a stably integrated copy of green fluorescent protein (GFP) whose expression is driven by the bradyzoite-specific LDH2 promoter, thereby facilitating identification of vacuoles containing bradyzoites (15). Genetic deletion of *Tg*PKAc3 by double homologous recombination-mediated integration of the hypoxanthine xanthine-guanine phosphoribosyltransferase (HXGPRT) selectable marker at the *Tg*PKAc3 locus was confirmed by PCR screening (Fig. 4B). The subsequent knockout strain was designated Pru*Δku80Δpkac3*. This mutant was then complemented with *Tg*PKAc3-3HA under the control of its native promoter. Integration of the *Tg*PKAc3-3HA expression cassette in the complemented strain Pru*Δku80Δpkac3*::PKAc3-3HA was confirmed by PCR (Fig. 4B). The immunofluorescence assay (IFA) was performed using anti-HA antibody, and bradyzoite differentiation was monitored by examining parasites for GFP expression (Fig. 4C). *Tg*PKAc3-3HA was expressed in both tachyzoites (GFP-negative parasites) and bradyzoites (GFP-positive parasites). The majority of the signal was in the parasite cytosol in tachyzoites, and punctate signals in the parasite periphery were observed only in bradyzoites. These distributions are consistent with what was seen with transient expression of these genes in *T. gondii* (Fig. 1B and 2B).

Immunoblot analysis demonstrated four distinct bands (Fig. 4D, four arrows) in the complemented strain Pru*Δku80Δpkac3*::PKAc3-3HA, which migrated close to the expected molecular mass of *Tg*PKAc3-3HA of 60.5 kDa (the 3HA tag is ~3.3 kDa). These bands may represent splicing variants, posttranslational modifications, or proteolysis. In the total protein lysates from each strain, GFP signal was increased in the Pru*Δku80Δpkac3* strain (Fig. 4D), suggesting that loss of *Tg*PKAc3 results in an increased proportion of bradyzoites under normal *in vitro* culture conditions (i.e., pH 7.2 and 5% CO_2_).

### The *Tg*PKAc3 mutant demonstrates a growth defect.

**FIG 5:**
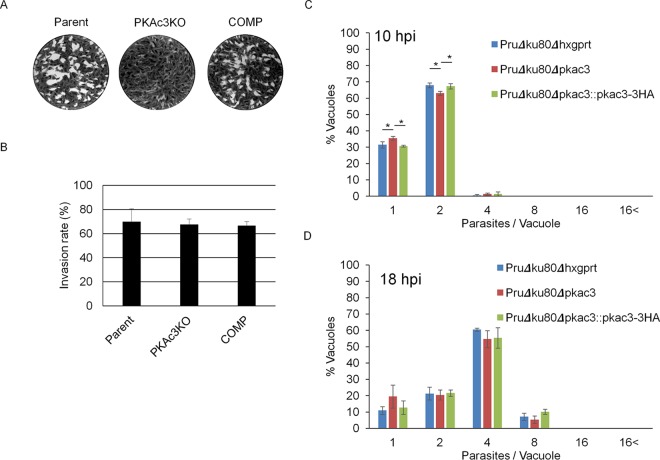
Growth of the *Tg*PKAc3 knockout under normal culture conditions. (A) Overall growth speed was measured by plaque assay. Forty parasites for Pru*Δku80Δhxgprt* and Pru*Δku80Δpkac3*::PKAc3-3HA or 80 parasites for Pru*Δku80Δpkac3* were inoculated into HFF and incubated for 14 days. (B) Parasites were allowed to invade the host cells for 30 min and fixed, and extracellular parasites and total parasites were stained sequentially. Invaded parasites/total parasites are shown as invasion rate. (C and D) Parasite numbers within vacuoles were counted at 10 h (C) or 18 h (D) after inoculation. Ratios of parasite number to vacuole are shown. If one-way analysis of variance detected a significant difference within parent, *Δpkac3*, and complemented strain values, then Tukey’s honestly significant difference test results are shown. *, *P* < 0.05; **, *P* < 0.01.

To measure the effect of *Tg*PKAc3 disruption on parasite growth throughout multiple cycles of replication, we performed plaque assays. Plaques from the Pru*Δku80Δpkac3* strain were not visible in this assay, and this mutant exhibited a marked growth defect compared to the parental strain (Fig. 5A), which was restored by genetic complementation with PKAc3-3HA in the Pru*Δku80Δpkac3*::PKAc3-3HA strain. PKA signaling is reported to have a role in host cell invasion (7, 8), and therefore, invasion efficiency was measured. There was no significant difference in invasion rates among the parental, null, and complemented strains (Fig. 5B). Next, parasite replication was examined, as PKA signaling has also been reported to regulate replication (6, 16). The parasite number within a parasitophorous vacuole at 10 h was slightly decreased in the *Δpkac3* strain and was recovered by complementation (Fig. 5C), whereas at 18 h postinfection (hpi), we could not find any significant differences in replication (Fig. 5D). This result suggests that the *Δpkac3* strain did not have a defect in invasion but might have had a defect in the early initiation of cell division, explaining the decrease in parasite number at the early time point. The cell division speed of *Δpkac3* strain was not different from that of the wild type, and thus, the difference between the parental strain and the *Δpkac3* strain did not increase over time.

### *Tg*PKAc3 knockout affects tachyzoite-to-bradyzoite differentiation in both type I and II *T. gondii* strains.

**FIG 6:**
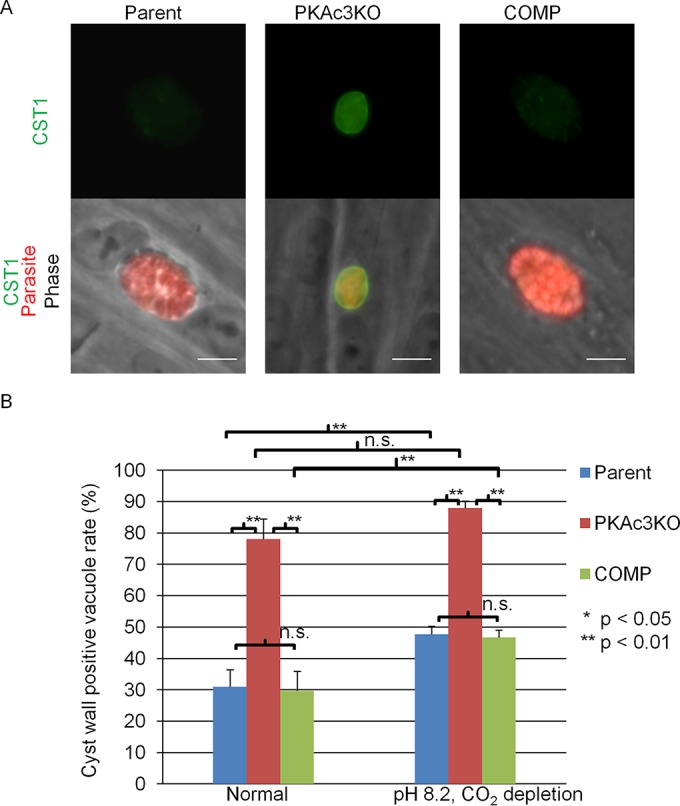
Disruption of *Tg*PKAc3 induces bradyzoite-specific cyst wall formation. The cyst wall was stained to measure the bradyzoite differentiation status in the parental parasite Pru*Δku80Δhxgprt* (Parent), knockout clone Pru*Δku80Δpkac3* (PKAc3KO), and complemented clone Pru*Δku80Δpkac3*::PKAc3-3HA (COMP). Infected host cells were grown under normal culture conditions or bradyzoite induction conditions (pH 8.2 and CO_2_ depletion) for 48 h. Parasites were stained with anti-CST1 antibody (shown in green) or anti-*Toxoplasma* serum (shown in red). (A) Representative images of vacuoles with cyst walls (center panel, Pru*Δku80Δpkac3*) and without cyst walls (left and right panels, showing Pru*Δku80Δhxgprt* and Pru*Δku80Δpkac3*::PKAc3-3HA, respectively) under tachyzoite culture condition. (B) Cyst wall-positive vacuoles were identified as parasitophorous vacuoles that have anti-CST1 signal associated with the parasitophorous vacuole. Quantitative measurements of the cyst wall-positive vacuole rate are shown. At least 100 total vacuoles per sample were counted, and cyst wall-positive vacuoles per total vacuoles are shown. Mean values and standard deviations from independent triplicate experiments are shown. If one-way analysis of variance detected significant differences among the group, Tukey’s honestly significant difference test results are shown. *, *P* < 0.05; **, *P* < 0.01; n.s., nonsignificant.

To determine whether *Tg*PKAc3 was involved in tachyzoite and bradyzoite regulation, we examined the rate of bradyzoite differentiation at 48 h postinfection. Pru*Δku80Δpkac3* had cyst wall staining even under normal culture conditions (Fig. 6A). The basal rate of bradyzoite formation in the mutant was much higher than the two control strains (80% versus 30%) when cultured under standard cell culture conditions (pH 7.2, 5% CO_2_) (Fig. 6B). When the CO_2_ concentration was dropped to 0.5% and the strains were cultured in pH 8.2 medium, the number of cyst wall-positive vacuoles increased in both the parental and mutant strains (Fig. 6B). Because Pru*Δku80Δpkac3* has a high differentiation rate even under normal culture conditions, it was difficult to tease out if the ablation of *Tg*PKAc3 affected bradyzoite induction by high pH and 0.5% CO_2_. Therefore, we next examined the effect of *Δpkac3* in the RH*Δku80Δhxgprt T. gondii* strain, which rarely differentiates into bradyzoites *in vitro* and would not be expected to have a high rate of bradyzoite induction under bradyzoite culture conditions (pH 8.2, 0.5% CO_2_).

**FIG 7:**
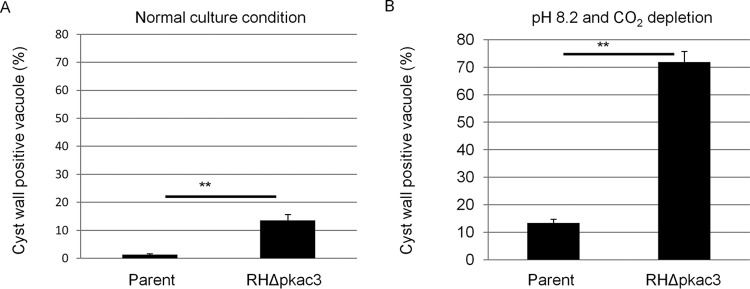
*Tg*PKAc3 affects bradyzoite differentiation in the RH strain. The cyst wall was stained to measure the bradyzoite differentiation status in the parental parasite RH*Δku80Δhxgprt* (Parent) and knockout clone RH*Δku80Δpkac3* (RH *Δpkac3*). Infected host cells were grown under normal culture conditions (A) or bradyzoite induction conditions (pH 8.2 and complete CO_2_ depletion) (B) for 48 h. Parasites were stained with anti-CST1 antibody and anti-*Toxoplasma* serum. Cyst wall-positive vacuoles were identified as parasitophorous vacuoles that have anti-CST1 signal associated with the parasitophorous vacuole. Quantitative measurements of the cyst wall-positive vacuole rate are shown. At least 100 total vacuoles per sample were counted, and cyst wall-positive vacuoles per total vacuoles are shown. Mean values and standard deviations from independent triplicate experiments are shown. Statistical differences determined by Student’s *t* test between parent and *Tg*PKAc3KO are shown (**, *P* < 0.01).

The RH*Δku80Δpkac3* strain was established (see Fig. S2A in the supplemental material) using the same strategy used for the Pru*Δku80Δpkac3* strain. RH*Δku80Δpkac3* plaques were detectable (see Fig. S2B), but the plaque sizes were significantly smaller than those of parental RH*Δku80Δhxgprt* (see Fig. S2B). Similarly to Pru*Δku80Δpkac3*, the RH*Δku80Δpkac3* strain did not show any difference in the invasion rate and did not have a difference in its rate of cell division (see Fig. S2C and D). The RH*Δku80Δpkac3* strain demonstrated cyst wall staining under normal culture conditions (~14% in RH*Δku80Δpkac3*), whereas the parental strain had few CST1-positive vacuoles (~1% in parental RH*Δku80Δhxgprt*) (Fig. 7A). To observe the effect of the bradyzoite induction in the RH strain, we treated cells with high pH without addition of CO_2_ (ambient air contains ~0.02% CO_2_). When the parasites were cultured under these bradyzoite induction conditions, 72% of RH*Δku80Δpkac3* vacuoles were cyst wall positive, significantly greater than that seen in the parental RH*Δku80Δhxgprt* (13%) (Fig. 7B). These results are consistent with *Tg*PKAc3 functioning as a negative regulator of bradyzoite differentiation under the high-pH, low-CO_2_ stress conditions widely used for bradyzoite research. This also demonstrated that the function of *Tg*PKAc3 is independent of strain lineage in *T. gondii*.

### cAMP-dependent tachyzoite maintenance is dependent on a *Tg*PKAc3 signal.

**FIG 8:**
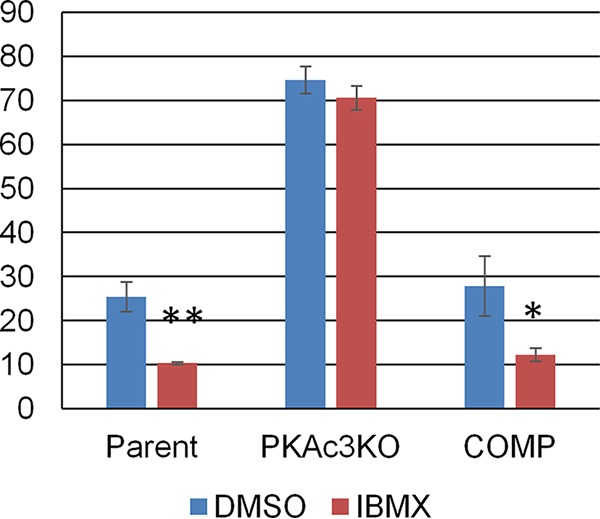
Long-term elevation of cAMP causes tachyzoite maintenance via *Tg*PKAc3 signal. After a 2-h invasion window, parasites were treated with IBMX (500 µM final concentration) or vehicle control DMSO for 48 h. The cyst wall was stained to measure the bradyzoite differentiation status in the parental parasite Pru*Δku80Δhxgprt* (Parent), knockout clone Pru*Δku80Δpkac3* (PKAc3KO), and complemented clone Pru*Δku80Δpkac3*::PKAc3-3HA (COMP). Cyst wall-positive vacuoles were identified as parasitophorous vacuoles that have anti-CST1 signal associated with the parasitophorous vacuole as shown in Fig. 5A. At least 100 total vacuoles per sample were counted, and percentages of cyst wall-positive vacuoles per total vacuoles are shown. Mean values and standard deviations from independent triplicate experiments are shown. Student’s *t* test between DMSO and IBMX treatment was performed. *, *P* < 0.05; **, *P* < 0.01.

According to previous studies, a prolonged elevation of cAMP in *T. gondii* maintains the growth of tachyzoites and prevents bradyzoite differentiation. Because the *Tg*PKAc3 mutant had a defect in maintaining tachyzoites under normal culture conditions, the involvement of *Tg*PKAc3 in cAMP-dependent tachyzoite maintenance was examined. The various *T. gondii* parental and mutant strains were treated with 500 µM 3-isobutyl-1-methylxanthine (IBMX), which is a phosphodiesterase inhibitor that causes a prolonged elevation of cAMP levels in *T. gondii* (6) and can cause bradyzoite repression (12). Consistent with our previous study on IBMX (6), cyst wall-positive vacuoles in wild-type parental *T. gondii* treated with IBMX decreased compared to dimethyl sulfoxide (DMSO) control-treated parasites. The *Δpkac3* strain did not have a change in its differentiation rate with IBMX treatment (Fig. 8). The *Tg*PKAc3 complementation restored the bradyzoite repression effect of IBMX (Fig. 8). These data suggest that cAMP-dependent tachyzoite maintenance utilizes *Tg*PKAc3 signaling.

### Effect of the absence of *Tg*PKAc3 on cell cycle regulation.

Bradyzoite differentiation is linked to regulation of the cell cycle in *T. gondii* (17). To further investigate how *T*gPKAc3 signaling could affect bradyzoite differentiation regulation, we characterized parasite cell cycle regulation in our mutants, and we quantified the population of parasites in the G_1_ phase during the first (8 h postinvasion) and second (16 h postinvasion) division cycles. We used a centrosome marker to estimate G_1_ versus S/M/C distributions to analyze asynchronous populations of parental and PKA-knockout strains (18). There was no difference in length of G_1_ or fractional distribution of M/C stages in the *Δpkac3* strain compared to the parental or *Tg*PKAc3-complemented *T. gondii* strain (see Fig. S3 in the supplemental material). Therefore, it appears that bradyzoite differentiation in the *Δpkac3* strain is not related to a specific defect in cell cycle regulation in this knockout strain.

### Effects of *Tg*PKAc3 deletion on parasite infection *in vivo.*

**FIG 9:**
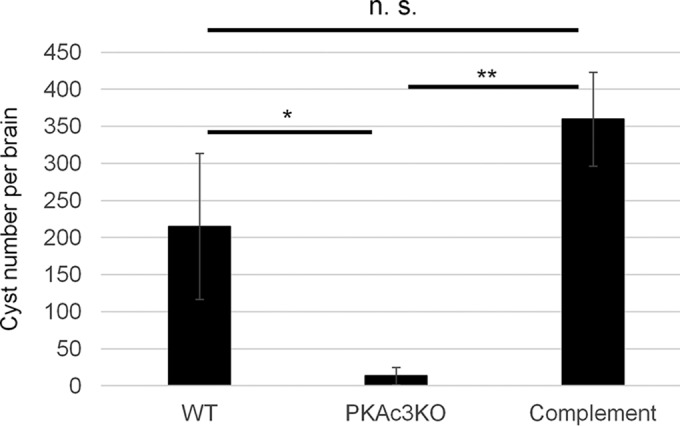
*In vivo* cyst production with *Tg*PKAc3 mutant parasites. Six- to 8-week-old female C57BL/6J mice were infected with 10,000 parasites via the intraperitoneal route. Seven weeks after infection, the tissue cyst number in the infected mouse brain was counted. The average numbers of cysts and standard deviations (*n* = 4 for wild type [WT], *n* = 3 for knockout [KO], and *n* = 4 for complemented) are shown. Mean values were statistically analyzed with one-way analysis of variance for detecting a difference in the groups and with a *post hoc* Tukey honestly significant difference test for pairwise comparison. n.s., *P* > 0.05; *, *P* < 0.05; **, *P* < 0.01.

The effects of *Tg*PKAc3 in the acute phase of infection were examined by infecting both RH- and Pru-derived *Tg*PKAc3 mutant strains and performing a survival curve analysis. Under all conditions tested, the absence of *Tg*PKAc3 did not significantly alter the survival curve of infected mice (see Fig. S4A in the supplemental material). To evaluate the effect of *Tg*PKAc3 on *in vivo* chronic infection, C57BL/6J mice were infected with the Pru-derived *Tg*PKAc3 strains. Brain cysts were quantified in survivor mouse brains at 7 weeks postinfection. The parental Pru*Δku80Δhxgprt*, *Δpkac3*, and *Tg*PKAc3-complemented strains all produced brain cysts (Fig. 9). Cyst number decreased in the Pru*Δku80Δpkac3* strain and returned to parental levels in the complemented strain (Fig. 9). These data suggest that ablation of *Tg*PKAc3 reduces the cyst number in the mouse brain during latent infection.

## DISCUSSION

Activation of PKA by a short-term cAMP pulse induces bradyzoite differentiation, whereas a prolonged cAMP pulse inhibits differentiation (7). It is likely that there are distinct PKA signaling pathways in the tachyzoite with opposing effects on parasite differentiation. Inhibition of PKA signals by treatment with PKA catalytic subunit inhibitor H89 induces bradyzoite differentiation (11), suggesting that PKA catalytic subunit activity may be involved in cAMP-mediated tachyzoite maintenance. The PKA catalytic isoform responsible for this effect could not be identified, as H89 treatment inhibits multiple PKA catalytic subunit isoforms. Our study provides the first insight into the mechanism by which cAMP signaling can regulate both bradyzoite induction and tachyzoite maintenance. The divergent PKA isoform *Tg*PKAc3 has a unique biological role in *T. gondii* in cAMP-dependent tachyzoite maintenance.

### Bradyzoite gene expression and cell division regulation are distinct events.

Unlike its parental strain, the *Tg*PKAc3 mutant does not exhibit coregulation of cell division and bradyzoite gene expression. Previous studies with wild-type *T. gondii* have shown that *in vivo* tissue cyst and *in vitro* stress-induced bradyzoites have concurrent bradyzoite gene expression and cell division slowing (16, 19), implying a cell cycle regulation different from that of tachyzoites (17). Although we observed increased expression of bradyzoite differentiation markers (cyst wall and GFP signal driven by bradyzoite-specific LDH2 promoter) under normal tachyzoite growth conditions, the cell division rate of Δ*pkac3* parasites after invasion was not significantly different from that of the parental or complemented strains. Cell cycle analysis also detected no differences between wild type and mutants (see Fig. S3 in the supplemental material) (17). The *Δpkac3* strain did not have the clear G_0_/G_1_ peak seen in fully mature bradyzoites from *in vivo* tissue cysts (17).

Collectively, these data suggest that the *Δpkac3* strain lacks the coordination between bradyzoite gene expression and cell division regulation that has been previously described. We propose a new bradyzoite differentiation model in which bradyzoite gene expression and cell cycle regulation are separately, but coordinately, regulated and integrated by specific signals, including the *Tg*PKAc3 signal. Proper cAMP signaling appears to be important for switching between tachyzoites and bradyzoites. The *Δpkac3* strain had fewer brain cysts, which could imply that integrated bradyzoite differentiation is also required for efficient production or maintenance of the cyst number *in vivo* in the mouse brain and other tissues. Stage-specific antigen expression regulation is known to regulate the immunogenicity of the bradyzoite antigens (20). The effects of antigen presentation from early bradyzoite gene expression during dissemination may be involved in the reduction of cyst numbers seen in the *Δpkac3* strain. Alternatively, *Δpkac3* strains may not readily disseminate to the brain.

Examination of published phosphoproteome data (available on http://www.ToxodB.org) for proteins with PKA motif [R/K] [R/K]X^phos^[S/T] (see full gene identifier [ID] list in Table S1 in the supplemental material) reveals several AP2 transcription factor family members with PKA phosphorylation sites, including AP2XI-4, which has been previously described as a regulator of bradyzoite gene expression (21). Enzymes involved in epigenetic modification, such as *Tg*GCN5b (TGGT1_243440) (22) and candidate histone lysine methyltransferases (TGGT1_000420, TGGT1_054420, and TGGT1_087910) also have phosphorylated PKA motifs (see Table S1). These putative PKA substrates suggest that parasite PKA signaling may regulate both transcription and chromatin structure to regulate genes important for maintenance of the tachyzoite state. PKA regulation of developmental transitions is a universal theme in eukaryotic biology, and PKA signaling in the budding yeast transduces the PKA catalytic subunit-specific signals by targeting a transcriptional factor (9).

### Possible regulatory mechanisms of PKA in *Toxoplasma*.

Both *Tg*PKAc1 and *Tg*PKAc3 have *in vitro* cAMP-dependent protein kinase activity. This suggests that both proteins have a capacity to interact with canonical PKAr (i.e., the mammalian PKAr in HEK293T cells which expressed the parasite PKAc). *Tg*PKAc1 expressed in wheat germ cell extract has been reported to be less susceptible to PKI and H89 than bovine PKA expressed in wheat germ extract (11). We were unable to produce active PKAc in bacterial systems. Due to the known issues in expressing fully active recombinant PKA, the *in vitro* kinase activity of immunoprecipitated *Tg*PKAc complex may differ from recombinant protein expressed *in vitro* using wheat germ cell extract. While the recombinant protein kinase domain of *Tg*PKAc3 was demonstrated to be active using an *in vitro* kinase assay, *Tg*PKAc3 has a large N-terminal domain that may be involved in the function of *Tg*PKAc3. Notably, *Tg*PKAc3 demonstrated a change in localization between tachyzoite (primarily diffuse in the cytosol) and bradyzoite (punctate distribution in the periphery of the parasite) conditions. Localization changes in PKA catalytic subunits have been reported, as a regulatory system for PKA activity in mammalian cells and localization of the PKA complex to subcompartments in cells are known to be regulated by diverse members of A kinase anchor protein (AKAP) (23). Although *T. gondii* lacks obvious AKAP homologs, Apicomplexa-specific atypical scaffolding proteins could act to regulate PKA activity. Overexpression of *Tg*PKAc3 caused a delay in tachyzoite replication (K. Kim and M. S. Eaton, unpublished data), also suggesting that regulation of the activity of *Tg*PKAc3 is required for proper cell cycle progression.

### Summary hypothesis.

We hypothesize that *Tg*PKAc3 functions to prevent bradyzoite gene expression and to keep tachyzoites replicating as tachyzoites. Several bradyzoite repressor genes in *Toxoplasma* have now been reported, including transcription factor AP2IX-9 (24) and *T. gondii* serine protease inhibitor 1 (*Tg*SPI1, TGME49_208450) (25). Both of these proteins repress bradyzoite differentiation under high-pH (8.2) conditions; hence, they are expected to function in early bradyzoite stages (22, 23). In contrast, the *Δpkac3* strain has high rates of bradyzoite formation even under the conditions that normally promote tachyzoite replication. Therefore, it is probable that *Tg*PKAc3 inhibits bradyzoite differentiation constitutively by maintaining tachyzoites. Spontaneous tachyzoite-to-bradyzoite differentiation and cell division speed changes have been noted using *in vitro* tissue cultures infected with VEG strain sporozoites (19), suggesting that bradyzoite differentiation is an innate programmed response and that spontaneous differentiation to bradyzoites is based on the number of cell divisions. In this model, the persistence of tachyzoites (i.e., inhibition of bradyzoites) is an active process that requires specific gene regulation and active signaling in the parasite. Further investigations of active tachyzoite maintenance signals are clearly needed to fully understand bradyzoite differentiation and regulation in this important apicomplexan.

## MATERIALS AND METHODS

### Sequence analysis.

*Tg*PKAc1 (TGME49_226030), *Tg*PKAc2 (TGME49_228420), and *Tg*PKAc3 (TGME49_286470), which were predicted to have an STKc-PKA-like domain (cd05580) with the NCBI conserved domain database (26), were retrieved from ToxoDB. Additional protein kinases were retrieved using a BLAST search employing *Tg*PKAc1, -2, and -3 as the query sequence. Genes of *Toxoplasma*, *Neospora*, and *Eimeria* were retrieved from ToxoDB. Genes of *Homo sapiens* and *Saccharomyces cerevisiae* (strain ATCC 204508/S288c) were retrieved from UniProt. Other genes were retrieved from NCBI GenBank. The protein kinase domain predicted with a conserved domain search (26) was used for alignment and phylogenetic analysis. Alignment and phylogenetic analysis were performed with MEGA6 (27). Phylogenetic relationships were then inferred by using the maximum likelihood method based on the Le-Gascuel 2008 model (28). An initial tree(s) for the heuristic search was obtained by applying the neighbor-joining method to a matrix of pairwise distances estimated using a JTT model. A discrete gamma distribution was used to model evolutionary rate differences among sites (5 categories [+*G*, parameter = 1.4834]). Amino acid positions that are conserved in more than 90% of proteins were used for calculation. There were a total of 287 positions in the final data set. Five thousand bootstrap replications were used to estimate the percentage of trees in which the associated taxa clustered together.

### Cell culture.

Human foreskin fibroblasts (HFF) were maintained in Dulbecco’s modified Eagle’s medium (DMEM; Life Technologies) supplemented with 10% fetal bovine serum (FBS), penicillin, and streptomycin (Life Technologies). The *T. gondii* RH*Δku80Δhxgprt* (29) strain was used in the overexpression study, and the Pru*Δku80Δhxgprt* strain, which expresses GFP driven by the bradyzoite-specific *LDH2* promoter (15), and RH*Δku80Δhxgprt* were used in the *Tg*PKAc3 knockout experiments. Parasites were cultured in HFF host cells using the same medium used for HFF culture using standard techniques (4).

### Overexpression of *Tg*PKAc1, -2, and -3.

Expression plasmids for *Tg*PKAc-1, -2, and -3 were constructed using the GRA1 promoter, and the cDNA of each gene was fused to a C-terminal HA tag. Five micrograms of each expression plasmid was transfected into RH*Δku80Δhxgprt* parasites and inoculated into HFF cells cultured on coverslips. The parasites were fixed at 24 h posttransfection. For the extracellular parasite, 24 h after transfection, parasites were harvested from the infected host cells and incubated in the culture medium at 37°C for 30 min before fixation. For the bradyzoite condition, transfected parasites were inoculated into HFF host cells, and 2 h after inoculation, medium was replaced with pH 8.2 medium and cells were cultured under a CO_2_-depletion condition for 2 days before fixation. Fixed cells were stained as described in the immunofluorescence assay section with anti-HA monoclonal antibody (MAb) 3F10 (Roche, Basel, Switzerland), rabbit anti-GAP45 antibody (30) (a generous gift from Dominique Soldati) in a 1:3,000 dilution, anti-IMC1 rabbit antibody (a generous gift from Michael White) in a 1:250 dilution, and anti-GFP rabbit antibody (Life Technologies, Carlsbad, CA) in a 1:500 dilution.

### Heterologous expression of kinase domain of *Tg*PKAc1 and -3.

C-terminally HA-tagged *Tg*PKAc1 coding sequence and the kinase domain of *Tg*PKAc3 (*Tg*PKAc3*Δ*120) were cloned into the pcDNA3 mammalian expression vector. Two micrograms of DNA was used to transfect 293T cells cultured in a 6-well plate with FuGene (Roche). Transfected cells were harvested 48 h later to purify the HA-tagged PKAc. Three wells of transfected 293T cells were lysed in 1.5 ml lysis buffer (150 mM NaCl, 1% Triton X-100, 50 mM Tris HCl [pH 8.0], and protease inhibitor cocktail [Roche]) for 30 min on ice. Cells were centrifuged for 10 min at 13,000 rpm, and supernatants were used as lysates. One hundred fifty microliters of anti-HA antibody conjugated to magnetic beads (Miltenyi Biotec, San Diego, CA) was added to cell lysates and incubated on ice for 30 min. Antibody-bound fraction was purified according to the manufacturer’s instruction with the μMACS isolation kit (Miltenyi Biotec). The protein kinase assay was performed with the protein bound to the column directly with a protein kinase A assay kit (Millipore, Billerica, MA). The reaction was carried out in a 25-µl reaction volume at 37°C for 10 min and stopped by spotting the reaction mixture onto P81 phosphocellulose paper. Filter paper was extensively dried for 30 s, washed 3 times with 0.75% H_3_PO_4_, and washed once with acetone. Incorporation of radioactive ^32^P to the phosphorylated PKA substrate peptide Kemptide was measured by scintillation counter.

### *Tg*PKAc3 knockout and complementation.

The knockout construct was built as previously described (31). Briefly, 1-kbp upstream and downstream genomic DNA sequences of *Tg*PKAc3 (TGME49_286470) were amplified from the genomic DNA from Pru*Δku80Δhxgprt.* These fragments were concatenated into pRS416 yeast shuttle vector flanking the selectable marker hypoxanthine xanthine-guanine phosphoribosyltransferase (*HXGPRT*) cassette using *S. cerevisiae* ATCC 90845 to make a ΔPKAc3 vector. All the primers used in the plasmid construct are listed in Table 1. *Toxoplasma gondii* PruΔ*ku80*Δ*hxgprt* was transfected with linearized ΔPKAc3 vector as previously described (31) and selected in the presence of 25 µg/ml mycophenolic acid and 50 µg/ml xanthine. Integration of HXGPRT selectable marker at the *Tg*PKAc3 locus was verified by PCR, and the parasites were subcloned by limiting dilution. Confirmation of the knockout was done using primer pairs detecting integration sites (KO_F and KO_R) and primers detecting the wild-type locus (WT_F and WT_R) as depicted in Fig. 4A. For the knockout of *Tg*PKAc3 in RH*Δku80Δhxgprt*, the same strategy was used, except that the knockout plasmid was prepared from the genomic DNA (gDNA) of RH*Δku80Δhxgprt* (see Text S1 in the supplemental material)*.*

**TABLE 1:** Primers used in the present study

Primer name	Sequence (5′→3′)^a^	Research objective
TGME49_286470- 5UTR_F	TTGGGTAACGCCAGGGTTTTCCCAGTCACGACGGTTTAAACCACGAACTAGACATAGACAGAGCCT	KO vector
TGME49_286470- 5UTR_R	GCGGGTTTGAATGCAAGGTTTCGTGCTGATCAAACTAGTCCAGTGGCCTGTCCCTGATGCACAT	KO vector
TGME49_286470- 3UTR_F	TTCTGGCAGGCTACAGTGACACCGCGGTGGAGGACTAGTTGAGCGCAAGGTGGTAGGCAGGTCT	KO vector
TGME49_286470- 3UTR_R	GTGAGCGGATAACAATTTCACACAGGAAACAGCGAAGCGCATACGAGCAAAGAGGAGTC	KO vector
KO_F	AGAGAAGACCTCTCGCCAAG	KO check
KO_R	ACTGCGAACAGCAGCAAGAT	KO check
WT_F	AGAGGAATAGACGGGAAAGA	WT check
WT_R	GAATGGGTTGGTGGATGAGA	WT check
COMP_F	TGATTACGCCAAGCTCGGAAAGAGAAGACCTCTCGCCAAG	Complement vector, complement check
CDS_R	CGTCGTACGGGTACCTAAAATTGTCGAAGAAGGCCTGCTG	Complement vector
pLIC-3HA-F	AGGTACCCGTACGACGTC	Complement vector
pLIC-3HA-R	TTCCGAGCTTGGCGTAATCA	Complement vector
COMP_R	CACCACTTCTCGTACTATGGC	Complement check

a Underlined sequences were used for ligation-independent cloning.

For the complementation of the *Tg*PKAc3-KO strain, a 4-kbp genomic locus of *Tg*PKAc3 including 2,279 bp upstream from the predicted start codon and the whole coding sequence except a stop codon was amplified from the gDNA of PruΔ*ku80*Δ*hxgprt* and cloned into the pLIC-3HA-DHFR plasmid (generous gift of Michael White), to produce a C-terminal 3HA fusion, *Tg*PKAc3, which was designated pPKAc3-3HA-DHFR (Fig. 3A). Five micrograms of pPKAc3-3HA-DHFR was linearized with the NotI restriction enzyme and nucleofected as described previously (32). Nucleofected parasites were selected with 1 µM pyrimethamine and subcloned by limiting dilution. The presence of the expression cassette for *Tg*PKAc3-3HA was confirmed with PCR with primers COMP_F and COMP_R.

### Immunofluorescence assay (IFA).

HFF cells grown on coverslips were infected with *T. gondii* and incubated for 48 h. Infected host cells were rinsed with ice-cold phosphate-buffered saline (PBS) three times and fixed with 4% paraformaldehyde in PBS for 20 min at room temperature. Fixed cells were treated with PBS containing 0.2 M glycine and 0.2% Triton X-100 for 20 min to quench the fixation and to permeabilize the membrane. Cells were washed with PBS three times and blocked with 2% bovine serum albumin in PBS with 0.1% Tween 20 (blocking buffer) for 60 min at 37°C. Antibodies were diluted in the same blocking buffer and incubated for 60 min at 37°C followed by washing three times with PBS with 0.1% Tween 20 (PBS-T). Rat monoclonal antibody 3F10 (Roche), which is a primary antibody for the HA epitope tag, was used at a 1:250 dilution. Alexa 594-conjugated anti-rat IgG goat secondary antibody (Life Technologies) was used at a 1:1,000 dilution. To enhance GFP signal, GFP was stained with anti-GFP rabbit antibody at a 1:500 dilution and Alexa 488-conjugated anti-rabbit IgG goat secondary antibody (Life Technologies). For counterstaining of nuclei, 4′,6-diamidino-2-phenylindole (DAPI) was used. Coverslips were mounted with ProLong Gold (Life Technologies), and images were acquired using a Leica TCS SP5 confocal microscope (Leica, Wetzlar, Germany).

### Immunoblotting (Western blotting).

Purified parasites were lysed in SDS-PAGE sample buffer. SDS-PAGE was performed with proteins from 10^6^ parasites per lane, and parasites were separated with 12% acrylamide gels. Proteins transferred on a polyvinylidene difluoride (PVDF) membrane were detected with rat anti-HA monoclonal antibody (MAb) 3F10 conjugated with horseradish peroxidase (HRP) (Roche), anti-GRA1 mouse MAb 92.10 B (33), and anti-GFP rabbit MAb (Life Technologies). Anti-mouse or -rabbit IgG conjugated with HRP (GE Healthcare Life Science, Little Chalfont, United Kingdom) was used as a secondary antibody.

### Plaque assay.

HFF cells were seeded into 6-well plates and cultured to form a confluent monolayer. Purified parasites were inoculated onto host cells at a dilution of 40 parasites/well. For the Pru*Δku80Δpkac3*, 80 parasites/well were inoculated in addition to the usual 40 parasites/well to ensure that potentially less viable parasites could also produce plaques. Plates were incubated without disturbance for 14 days until the parasites formed a visible host lysis plaque. After incubation, cells were fixed and stained with 20% methanol-0.5% crystal violet solution, washed with water, and dried before imaging.

### Invasion assay.

Double staining of extracellular parasites and total parasites was performed as described elsewhere (34). Briefly, purified parasites were inoculated into HFF cells in a 24-well plate at a dilution of 10^6^ parasites/well in 500 µl medium and incubated for 10 min at room temperature and 30 min at 37°C. After incubation, medium was aspirated and cells were fixed with 4% paraformaldehyde for 20 min. After primary fixation, cells were washed with PBS three times and extracellular parasites were reacted with anti-*Toxoplasma* rabbit sera diluted 1:4,000 in PBS containing 2% fetal bovine serum (FBS), followed by washing three times in PBS and secondary fixation with 4% paraformaldehyde for 20 min at room temperature. After secondary fixation, cells were incubated with 0.2 M glycine and 0.2% Triton X-100 in PBS for 20 min to quench the fixation and permeabilize the membrane, followed by three PBS washes. Permeabilized cells were blocked with 3% skim milk in PBS-T for 60 min at 37°C, and total parasites were stained with anti-GRA1 mouse MAb 92.10 B diluted at 500:1 in the same blocking buffer. After three washes with PBS-T, DAPI-, Alexa 488-, or Alexa 594-conjugated anti-mouse or -rabbit secondary antibodies were diluted at 1,000:1 in the same blocking buffer and incubated for 60 min at 37°C followed by five PBS-T washes. Intracellular parasites per total parasites were calculated as the invasion rate.

### Assay for parasite replication in host cell.

Purified parasites were inoculated onto HFF cells in a 24-well plate at a dilution of 10^5^ parasites/well in 500 µl medium and incubated for 30 min at 37°C. After incubation, cells were washed with warm medium with pipetting and incubated with the same medium for 10 and 18 h extensively. The parasite inner membrane complex was detected with anti-IMC7 (TGME49_222220) polyclonal mouse antibody (L. Weiss, unpublished data). IMC7 reportedly localizes to mature mother cell inner membrane complex (35). At the same time, anti-GFP antibody was used to label the bradyzoite parasites. At least 100 total parasitophorous vacuoles were counted for one sample, and the number of parasites per vacuole was calculated.

### Bradyzoite differentiation assay.

Bradyzoite differentiation was measured by staining the cyst wall. Briefly, purified parasites were inoculated onto HFF cells in a 24-well plate at a concentration of 1,000 parasites/well. After a 2-h incubation, the medium was changed to fresh medium and incubated under a 5% CO_2_ condition (normal) or was changed to induction medium (DMEM supplemented with 1% FBS and 50 mM HEPES, adjusted to pH 8.2 with NaOH) and incubated under an 0.5% CO_2_ condition (pH 8.2 and CO_2_ depletion). For the bradyzoite induction with the RH strain, ambient air with a humid box was used; thus, CO_2_ concentration was around 0.02%. Infected host cells were extensively incubated for 48 h, fixed with 4% paraformaldehyde in PBS, and stained as described for the immunofluorescence assay. Salmon E monoclonal antibody (1:500) for cyst wall protein 1 (CST1) (31) and 1:4,000-diluted rabbit anti-*Toxoplasma* serum were used as primary antibodies to detect cyst wall and total parasites, respectively. Alexa 488- and 594-conjugated anti-mouse or -rabbit IgG antibody diluted 1:1,000 was used as a secondary antibody. Green fluorescent signal at the parasitophorous vacuole membrane was identified as the cyst wall. At least 100 vacuoles were scored to determine the number of parasitophorous vacuoles with or without cyst walls.

### Animal experiments.

Ten female 6- to 8-week-old C57BL/6J mice (The Jackson Laboratory, Bar Harbor, ME) per group were infected with purified tachyzoites intraperitoneally. For the brain cyst burden determination, at 7 weeks postinfection surviving mice in each group were euthanized and brains were collected for the brain cyst counting. Brains were cut in half, and one half was homogenized in PBS to a final volume of 600 µl for each brain. Samples of 60 µl were counted, and total cyst counts per brain were calculated.

### Ethics statement.

All animal experiments were conducted according to the United States Public Health Service Policy on Humane Care and Use of Laboratory Animals. Animals were maintained in an AAALAC-approved facility, and all protocols were approved by the Institutional Care Committee of the Albert Einstein College of Medicine, Bronx, NY (Animal Protocols 20121104, 20121109, and 20121110; Animal Welfare Assurance no. A3312-01). No human samples were used in these experiments. Human foreskin fibroblasts were obtained from the ATCC.

## SUPPLEMENTAL MATERIAL

Text S1  Supplemental materials and methods. Download Text S1, DOCX file, 0.1 MB

Figure S1  Localization of *Tg*PKAc3 and *Tg*PKAr. (A) Parental parasite PLK*Δhxgprt* and PLK*Δhxgprt*::PKAc3-3HA parasites were inoculated into host cells and incubated for 24 h before fixation. Fixed cells were stained with anti-HA rat monoclonal antibody followed by anti-rat Alexa 594 and anti-PKAr rabbit antisera (Kim and Eaton, unpublished) followed by anti-rabbit Alexa 488. Nuclei were stained with DAPI. Bar, 7.5 µm. Download Figure S1, TIF file, 1.9 MB

Figure S2  Characterization of RH*Δpkac3.* (A) An integration of the selectable marker at the native locus of *Tg*PKAc3 was confirmed by PCR as described in Fig. 2A and B. Genomic DNA purified from the parental strain RH*Δku80Δhxgprt* or RH*Δku80Δpkac3* was amplified with the primer set KO_F and KO_R or WT_F and WT_R to detect the integrated selectable marker or deletion of the wild-type locus, respectively. (B) Overall growth speed was measured by plaque assay. Ten parasites for RH*Δku80Δhxgprt* and RH*Δku80Δpkac3* were inoculated into HFF cells confluent in a 6-well plate and incubated for 12 days. Infected host cells were stained with crystal violet. Representative plaque image is shown. Plaque size was measured from independent duplicate plaque assays. Relative plaque size normalized with average plaque size from RH*Δku80Δhxgprt* plaques is shown. Average plaque size and standard errors from the whole observed plaques are shown (*n* = 12 from WT and *n* = 9 in KO). Statistical analysis was performed with Student’s *t* test, and a *P* value of <0.01 is shown as **. (C) Parasites were inoculated into host monolayers, and 30 min after incubation, infected host cells were fixed and double labeled with extracellular and intracellular parasites by the same methods as those for Fig. 4B. Invasion rates of parental RHΔ*ku80Δhxgprt* and RH*Δku80Δpkac3* are shown. Average values and standard deviations from three independent experiments are shown. Student’s *t* test detected no significant difference between the strains. (D) Parasite replication was measured at 10 and 18 h postinfection. At least 100 vacuoles were counted, and ratios of the vacuoles containing each parasite number are shown. Student’s *t* test detected no significant difference between the strains. Download Figure S2, TIF file, 0.5 MB

Figure S3  Cell cycle analysis detected no difference in the *Tg*PKAc3 mutant. The presence of G_1_-phase cells was evaluated during the first (8 h postinvasion) and second (16 h postinvasion) division cycles. Centrosome marker centrin 1 was used to estimate G_1_ versus S/M/C distributions to analyze asynchronous populations of parental and PKA knockout strains. Average values and standard deviations from three independent experiments are shown for the percentage of vacuoles with G_1_ parasites. Download Figure S3, TIF file, 0.1 MB

Figure S4  Survival curve of the *Tg*PKAc3 mutant-infected mice. Six- to 8-week-old female C57BL/6J mice were infected with numbers and strains indicated in the figure via the intraperitoneal route (A to D). Ten mice per group were infected and observed daily, and dead mice were recorded until 27 days postinfection. Statistical tests of differences between *Tg*PKAc3 mutants and the parental strain were done with the log rank test. Download Figure S4, TIF file, 0.2 MB

Table S1  PKA motif phosphorylated proteins in published phosphoproteomics data. The phosphoproteomics data set which was published by Treeck et al. (reference 1 in Text S1) was searched for the PKA substrate motif [R/K] [R/K]X^phos^[S/T] (reference 2 in Text S1). From these phosphoproteomics data, 632 phosphorylated proteins (of 3,051 total) present in purified extracellular parasite samples and 499 phosphorylated proteins (of 2,501 total) present in the intracellular parasite sample had a PKA substrate motif. The gene ID list was used to query ToxoDB to get annotations of the genes. Table S1, XLSX file, 0.1 MB

## References

[1] Pappas G, Roussos N, Falagas ME 2009 Toxoplasmosis snapshots: global status of *Toxoplasma gondii* seroprevalence and implications for pregnancy and congenital toxoplasmosis. Int J Parasitol 39:1385–1394. doi:10.1016/j.ijpara.2009.04.003. 19433092

[2] Dubey JP, Frenkel JK 1976 Feline toxoplasmosis from acutely infected mice and the development of Toxoplasma cysts. J Protozool 23:537–546. doi:10.1111/j.1550-7408.1976.tb03836.x. 1003342

[3] Sakikawa M, Noda S, Hanaoka M, Nakayama H, Hojo S, Kakinoki S, Nakata M, Yasuda T, Ikenoue T, Kojima T 2012 Anti-*Toxoplasma* antibody prevalence, primary infection rate, and risk factors in a study of toxoplasmosis in 4,466 pregnant women in Japan. Clin Vaccine Immunol 19:365–367. doi:10.1128/CVI.05486-11. 22205659 PMC3294603

[4] Weiss L, Kim K 2014 *Toxoplasma gondii*. The model apicomplexan—perspectives and methods, 2nd ed. Academic Press (Elsevier Inc.), London, United Kingdom.

[5] Ferreira da Silva MDF, Barbosa HS, Gross U, Lüder CGK 2008 Stress-related and spontaneous stage differentiation of *Toxoplasma gondii*. Mol Biosyst 4:824–834. doi:10.1039/b800520f. 18633484

[6] Kirkman LA, Weiss LM, Kim K 2001 Cyclic nucleotide signaling in *Toxoplasma gondii* bradyzoite differentiation. Infect Immun 69:148–153. doi:10.1128/IAI.69.1.148-153.2001. 11119500 PMC97866

[7] Hartmann A, Arroyo-Olarte RD, Imkeller K, Hegemann P, Lucius R, Gupta N 2013 Optogenetic modulation of an adenylate cyclase in *Toxoplasma gondii* demonstrates a requirement of the parasite cAMP for host-cell invasion and stage differentiation. J Biol Chem 288:13705–13717. doi:10.1074/jbc.M113.465583. 23525100 PMC3650408

[8] Kim C, Xuong N-H, Taylor SS 2005 Crystal structure of a complex between the catalytic and regulatory (RIalpha) subunits of PKA. Science 307:690–696. doi:10.1126/science.1104607. 15692043

[9] Robertson LS, Fink GR 1998 The three yeast A kinases have specific signaling functions in pseudohyphal growth. Proc Natl Acad Sci U S A 95:13783–13787. doi:10.1073/pnas.95.23.13783. 9811878 PMC24897

[10] Robertson LS, Causton HC, Young RA, Fink GR 2000 The yeast A kinases differentially regulate iron uptake and respiratory function. Proc Natl Acad Sci U S A 97:5984–5988. doi:10.1073/pnas.100113397. 10811893 PMC18545

[11] Kurokawa H, Kato K, Iwanaga T, Sugi T, Sudo A, Kobayashi K, Gong H, Takemae H, Recuenco FC, Horimoto T, Akashi H 2011 Identification of *Toxoplasma gondii* cAMP dependent protein kinase and its role in the tachyzoite growth. PLoS One 6:e00755-16. doi:10.1371/journal.pone.0022492. PMC3140512 21799871

[12] Eaton MS, Weiss LM, Kim K 2006 Cyclic nucleotide kinases and tachyzoite–bradyzoite transition in *Toxoplasma gondii*. Int J Parasitol 36:107–114. doi:10.1016/j.ijpara.2005.08.014. 16216248 PMC3109623

[13] Leykauf K, Treeck M, Gilson PR, Nebl T, Braulke T, Cowman AF, Gilberger TW, Crabb BS 2010 Protein kinase A dependent phosphorylation of apical membrane antigen 1 plays an important role in erythrocyte invasion by the malaria parasite. PLoS Pathog 6:e00755-16. doi:10.1371/journal.ppat.1000941. PMC2880582 20532217

[14] Hehl AB, Basso WU, Lippuner C, Ramakrishnan C, Okoniewski M, Walker RA, Grigg ME, Smith NC, Deplazes P 2015 Asexual expansion of *Toxoplasma gondii* merozoites is distinct from tachyzoites and entails expression of non-overlapping gene families to attach, invade, and replicate within feline enterocytes. BMC Genomics 16:66. doi:10.1186/s12864-015-1225-x. 25757795 PMC4340605

[15] Fox BA, Falla A, Rommereim LM, Tomita T, Gigley JP, Mercier C, Cesbron-Delauw M-F, Weiss LM, Bzik DJ 2011 Type II *Toxoplasma gondii* KU80 knockout strains enable functional analysis of genes required for cyst development and latent infection. Eukaryot Cell 10:1193–1206. doi:10.1128/EC.00297-10. 21531875 PMC3187049

[16] Watts E, Zhao Y, Dhara A, Eller B, Patwardhan A, Sinai AP 2015 Novel approaches reveal that *Toxoplasma gondii* bradyzoites within tissue cysts are dynamic and replicating entities in vivo. mBio 6:e00755-16. doi:10.1128/mBio.01155-15. PMC4600105 26350965

[17] Radke JR, Guerini MN, Jerome M, White MW 2003 A change in the premitotic period of the cell cycle is associated with bradyzoite differentiation in *Toxoplasma gondii*. Mol Biochem Parasitol 131:119–127. doi:10.1016/S0166-6851(03)00198-1. 14511810

[18] El Bissati K, Suvorova ES, Xiao H, Lucas O, Upadhya R, Ma Y, Angeletti RH, White MW, Weiss LM, Kim K 2016 *Toxoplasma gondii* arginine methyltransferase 1 (PRMT1) is necessary for centrosome dynamics during tachyzoite cell division. mBio 7:e00755-16. doi:10.1128/mBio.02094-15. 26838719 PMC4742710

[19] Jerome ME, Radke JR, Bohne W, Roos DS, White MW 1998 *Toxoplasma gondii* bradyzoites form spontaneously during sporozoite-initiated development. Infect Immun 66:4838–4844. 9746587 10.1128/iai.66.10.4838-4844.1998 PMC108598

[20] Kim S-K, Boothroyd JC 2005 Stage-specific expression of surface antigens by *Toxoplasma gondii* as a mechanism to facilitate parasite persistence. J Immunol 174:8038–8048. doi:10.4049/jimmunol.174.12.8038. 15944311

[21] Walker R, Gissot M, Croken MM, Huot L, Hot D, Kim K, Tomavo S 2013 The Toxoplasma nuclear factor TgAP2XI-4 controls bradyzoite gene expression and cyst formation. Mol Microbiol 87:641–655. doi:10.1111/mmi.12121. 23240624 PMC3556193

[22] Wang J, Dixon SE, Ting LM, Liu TK, Jeffers V, Croken MM, Calloway M, Cannella D, Ali Hakimi M, Kim K, Sullivan WJ, Hakimi MA, Kim K, Sullivan WJ 2014 Lysine acetyltransferase GCN5b Interacts with AP2 factors and is required for *Toxoplasma gondii* proliferation. PLoS Pathog 10:e00755-16. doi:10.1371/journal.ppat.1003830. PMC3879359 24391497

[23] Feliciello A, Gottesman ME, Avvedimento EV 2001 The biological functions of A-kinase anchor proteins. J Mol Biol 308:99–114. doi:10.1006/jmbi.2001.4585. 11327755

[24] Radke JB, Lucas O, De Silva EK, Ma Y, Sullivan WJ, Weiss LM, Llinas M, White MW 2013 ApiAP2 transcription factor restricts development of the Toxoplasma tissue cyst. Proc Natl Acad Sci U S A 110:6871–6876. doi:10.1073/pnas.1300059110. 23572590 PMC3637731

[25] Pszenny V, Davis PH, Zhou XW, Hunter CA, Carruthers VB, Roos DS 2012 Targeted disruption of *Toxoplasma gondii* serine protease inhibitor 1 increases bradyzoite cyst formation in vitro and parasite tissue burden in mice. Infect Immun 80:1156–1165. doi:10.1128/IAI.06167-11. 22202120 PMC3294639

[26] Marchler-Bauer A, Lu S, Anderson JB, Chitsaz F, Derbyshire MK, DeWeese-Scott C, Fong JH, Geer LY, Geer RC, Gonzales NR, Gwadz M, Hurwitz DI, Jackson JD, Ke Z, Lanczycki CJ, Lu F, Marchler GH, Mullokandov M, Omelchenko MV, Robertson CL, Song JS, Thanki N, Yamashita RA, Zhang D, Zhang N, Zheng C, Bryant SH 2011 CDD: a Conserved Domain Database for the functional annotation of proteins. Nucleic Acids Res 39:D225–D229. doi:10.1093/nar/gkq1189. 21109532 PMC3013737

[27] Tamura K, Stecher G, Peterson D, Filipski A, Kumar S 2013 MEGA6: molecular evolutionary genetics analysis version 6.0. Mol Biol Evol 30:2725–2729. doi:10.1093/molbev/mst197. 24132122 PMC3840312

[28] Le SQ, Gascuel O 2008 An improved general amino acid replacement matrix. Mol Biol Evol 25:1307–1320. doi:10.1093/molbev/msn067. 18367465

[29] Fox BA, Ristuccia JG, Gigley JP, Bzik DJ 2009 Efficient gene replacements in *Toxoplasma gondii* strains deficient for nonhomologous end joining. Eukaryot Cell 8:520–529. doi:10.1128/EC.00357-08. 19218423 PMC2669201

[30] Plattner F, Yarovinsky F, Romero S, Didry D, Carlier MF, Sher A, Soldati-Favre D 2008 Toxoplasma profilin is essential for host cell invasion and TLR11-dependent induction of an interleukin-12 response. Cell Host Microbe 3:77–87. doi:10.1016/j.chom.2008.01.001. 18312842

[31] Tomita T, Bzik DJ, Ma YF, Fox BA, Markillie LM, Taylor RC, Kim K, Weiss LM 2013 The *Toxoplasma gondii* cyst wall protein CST1 is critical for cyst wall integrity and promotes bradyzoite persistence. PLoS Pathog 9:e00755-16. doi:10.1371/journal.ppat.1003823. PMC3873430 24385904

[32] Sugi T, Kawazu S, Horimoto T, Kato K 2015 A single mutation in the gatekeeper residue in TgMAPKL-1 restores the inhibitory effect of a bumped kinase inhibitor on the cell cycle. Int J Parasitol Drugs Drug Resist 5:1–8. doi:10.1016/j.ijpddr.2014.12.001. 25941623 PMC4412912

[33] Matthiesen SH, Shenoy SM, Kim K, Singer RH, Satir BH 2001 A parafusin-related Toxoplasma protein in Ca^2+^-regulated secretory organelles. Eur J Cell Biol 80:775–783. doi:10.1078/0171-9335-00214. 11831391

[34] Sugi T, Kato K, Kobayashi K, Watanabe S, Kurokawa H, Gong H, Pandey K, Takemae H, Akashi H 2010 Use of the kinase inhibitor analog 1NM-PP1 reveals a role for *Toxoplasma gondii* CDPK1 in the invasion step. Eukaryot Cell 9:667–670. doi:10.1128/EC.00351-09. 20173034 PMC2863409

[35] Anderson-White BR, Ivey FD, Cheng K, Szatanek T, Lorestani A, Beckers CJ, Ferguson DJ, Sahoo N, Gubbels MJ 2011 A family of intermediate filament-like proteins is sequentially assembled into the cytoskeleton of *Toxoplasma gondii*. Cell Microbiol 13:18–31. doi:10.1111/j.1462-5822.2010.01514.x. 20698859 PMC3005026

