# Supplementary material for: Toxoplasma gondii Cyclic AMP-Dependent Protein Kinase Subunit 3 Is Involved in the Switch from Tachyzoite to Bradyzoite Development
Source: mBio. 2016 May 31;7(3):e00755-16. doi: 10.1128/mBio.00755-16 (PMC4895117; doi:10.1128/mBio.00755-16)
Supplement: Text S1 — Supplemental materials and methods. Download [file mbo003162842s1.docx]

**Supplemental Materials and Methods**

**Generation of the RH*Δku80Δpkac3***

To make the knock out plasmid, upstream and downstream region flanking *Tg*PKAc3 native locus was amplified from the genomic DNA of RH*Δku80Δhxgprt* with primer sets 286470_5F TCGAGCTCGGTAATTTAAATCACGAACTAGACATAGACAG and 286470_RH-5R TTCGTGCTGATCAAGGTACCTGTCCCTGATGCACAT, 286470_3F or GACACCGCGGTGGAGGTACCTGAGCGCAAGGTGGTAGGCA and 286470_3R TCTAGAGGATCCATTTAAATGAAGCGCATACGAGCAAAGA (underlined sequences are used for the Gibson assembly cloning system)*.* HXGPRT Selectable marker and plasmid backbone was amplified from the *Δ*PKAc3 vector and pUC19 vector with primers HXGPRT-KpnI-F, GGTACCTTGATCAGCACGAAACCTTGC and

HXGPRT-KpnI-R, GGTACCTCCACCGCGGTGTCA or pUC19productionF-SwaI, AAATGGATCCTCTAGAGTCGACCTGC and pUC19productionR-SwaI, AAATTACCGAGCTCGAATTCACTGG. The resultant four fragments were assembled with Gibson assembly (NEB, MA, USA) according to the manufacture’s instruction to make pDelta-RH286470-HXGPRT. The resultant plasmid was cut with *Swa*I to cut the plasmid backbone and 40 µg linearized DNA was transfected into RH*Δku80Δhxgprt* and selected and knock out clones were picked and screened as described in the section of generation of *Tg*PKAc3 knock out in Pru*Δku80Δhxgprt* in main text.

**References**

1. **Treeck M**, **Sanders JL**, **Elias JE**, **Boothroyd JC**. 2011. The Phosphoproteomes of Plasmodium falciparum and Toxoplasma gondii Reveal Unusual Adaptations Within and Beyond the Parasites’ Boundaries. Cell Host Microbe **10**:410–419.

2. **Giansanti P**, **Stokes MP**, **Silva JC**, **Scholten A**, **Heck AJR**. 2013. Interrogating cAMP-dependent kinase signaling in Jurkat T cells via a protein kinase A targeted immune-precipitation phosphoproteomics approach. Mol Cell Proteomics **12**:3350–9.
