# Supplementary figures and images for: Toxoplasma gondii Cyclic AMP-Dependent Protein Kinase Subunit 3 Is Involved in the Switch from Tachyzoite to Bradyzoite Development
Source: mBio. 2016 May 31;7(3):e00755-16. doi: 10.1128/mBio.00755-16 (PMC4895117; doi:10.1128/mBio.00755-16)

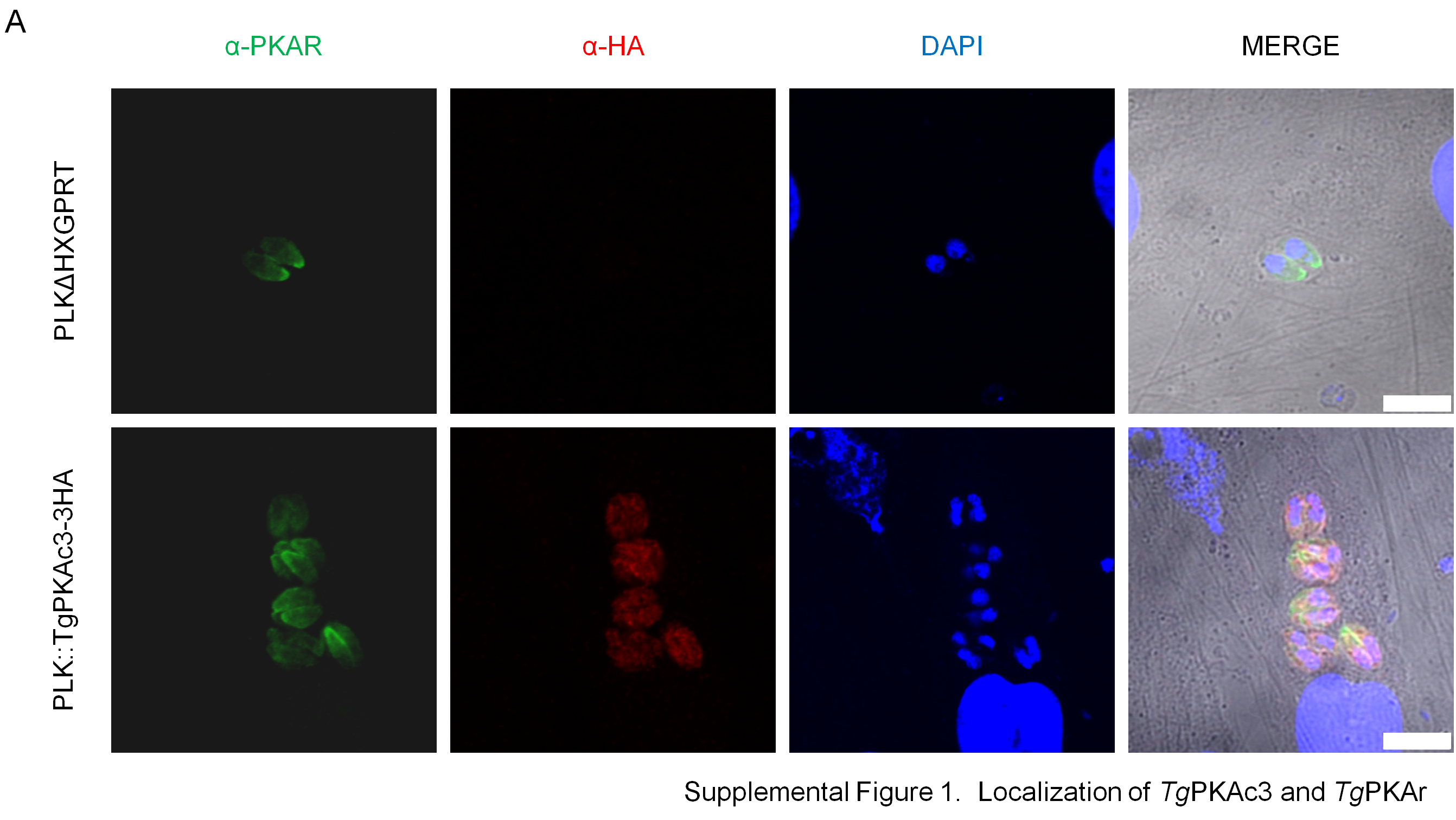

Supplement: Figure S1 — Localization of TgPKAc3 and TgPKAr. (A) Parental parasite PLKΔhxgprt and PLKΔhxgprt::PKAc3-3HA parasites were inoculated into host cells and incubated for 24 h before fixation. Fixed cells were stained with anti-HA rat monoclonal antibody followed by anti-rat Alexa 594 and anti-PKAr rabbit antisera (Kim and Eaton, unpublished) followed by anti-rabbit Alexa 488. Nuclei were stained with DAPI. Bar, 7.5 µm. Download [file mbo003162842sf1.tif]

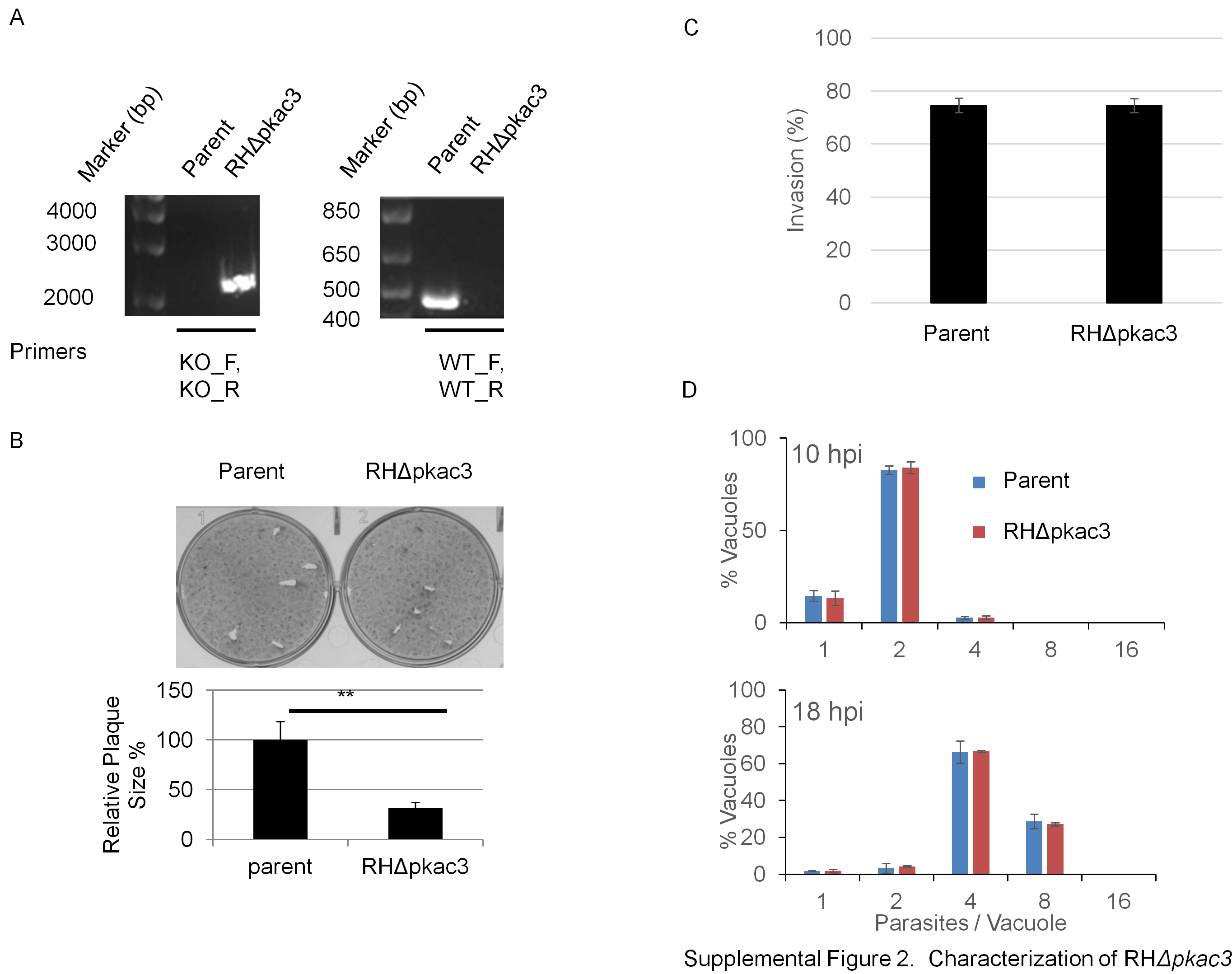

Supplement: Figure S2 — Characterization of RHΔpkac3. (A) An integration of the selectable marker at the native locus of TgPKAc3 was confirmed by PCR as described in Fig. 2A and B. Genomic DNA purified from the parental strain RHΔku80Δhxgprt or RHΔku80Δpkac3 was amplified with the primer set KO_F and KO_R or WT_F and WT_R to detect the integrated selectable marker or deletion of the wild-type locus, respectively. (B) Overall growth speed was measured by plaque assay. Ten parasites for RHΔku80Δhxgprt and RHΔku80Δpkac3 were inoculated into HFF cells confluent in a 6-well plate and incubated for 12 days. Infected host cells were stained with crystal violet. Representative plaque image is shown. Plaque size was measured from independent duplicate plaque assays. Relative plaque size normalized with average plaque size from RHΔku80Δhxgprt plaques is shown. Average plaque size and standard errors from the whole observed plaques are shown (n = 12 from WT and n = 9 in KO). Statistical analysis was performed with Student’s t test, and a P value of <0.01 is shown as **. (C) Parasites were inoculated into host monolayers, and 30 min after incubation, infected host cells were fixed and double labeled with extracellular and intracellular parasites by the same methods as those for Fig. 4B. Invasion rates of parental RHΔku80Δhxgprt and RHΔku80Δpkac3 are shown. Average values and standard deviations from three independent experiments are shown. Student’s t test detected no significant difference between the strains. (D) Parasite replication was measured at 10 and 18 h postinfection. At least 100 vacuoles were counted, and ratios of the vacuoles containing each parasite number are shown. Student’s t test detected no significant difference between the strains. Download [file mbo003162842sf2.tif]

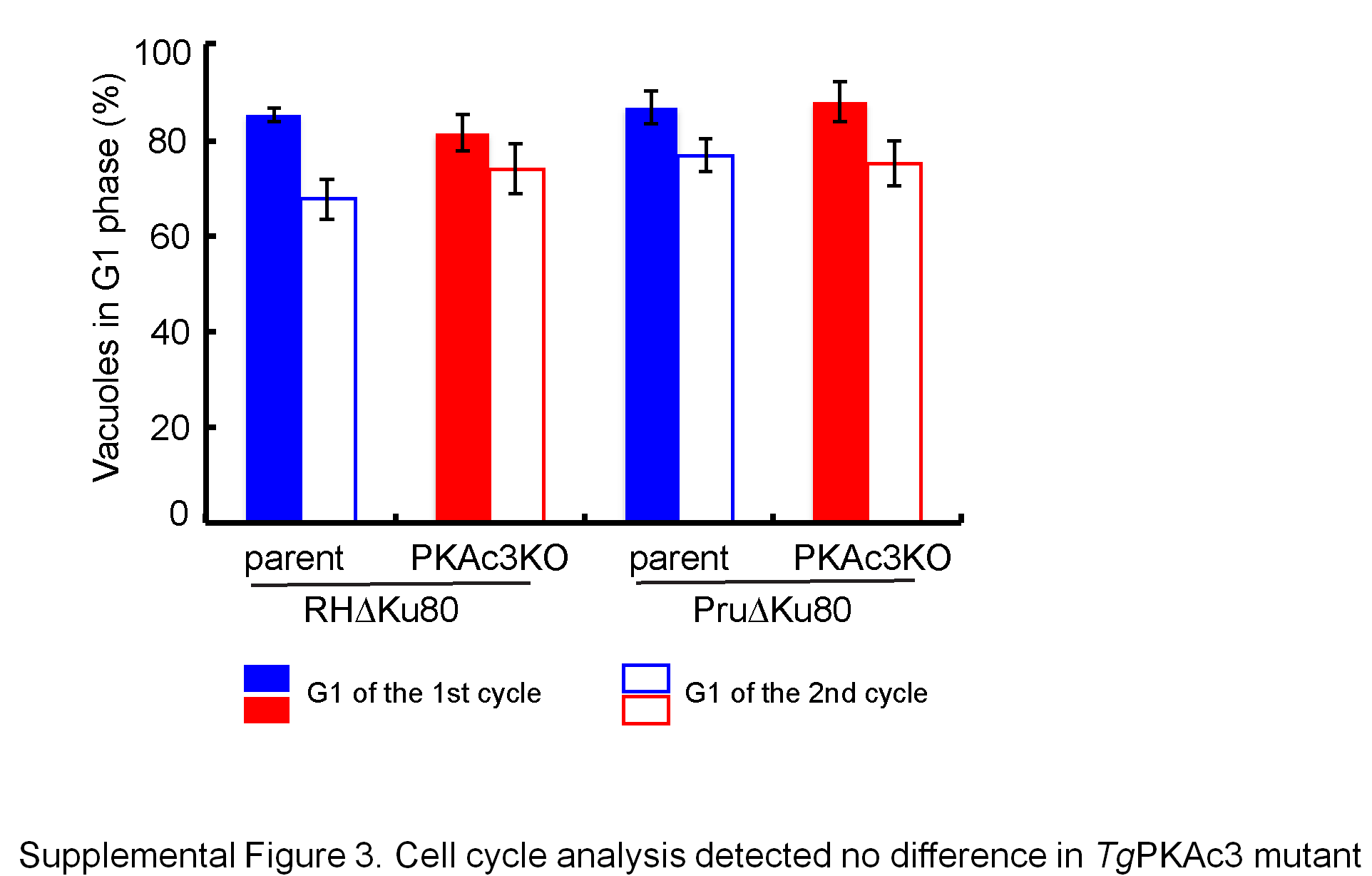

Supplement: Figure S3 — Cell cycle analysis detected no difference in the TgPKAc3 mutant. The presence of G1-phase cells was evaluated during the first (8 h postinvasion) and second (16 h postinvasion) division cycles. Centrosome marker centrin 1 was used to estimate G1 versus S/M/C distributions to analyze asynchronous populations of parental and PKA knockout strains. Average values and standard deviations from three independent experiments are shown for the percentage of vacuoles with G1 parasites. Download [file mbo003162842sf3.tif]

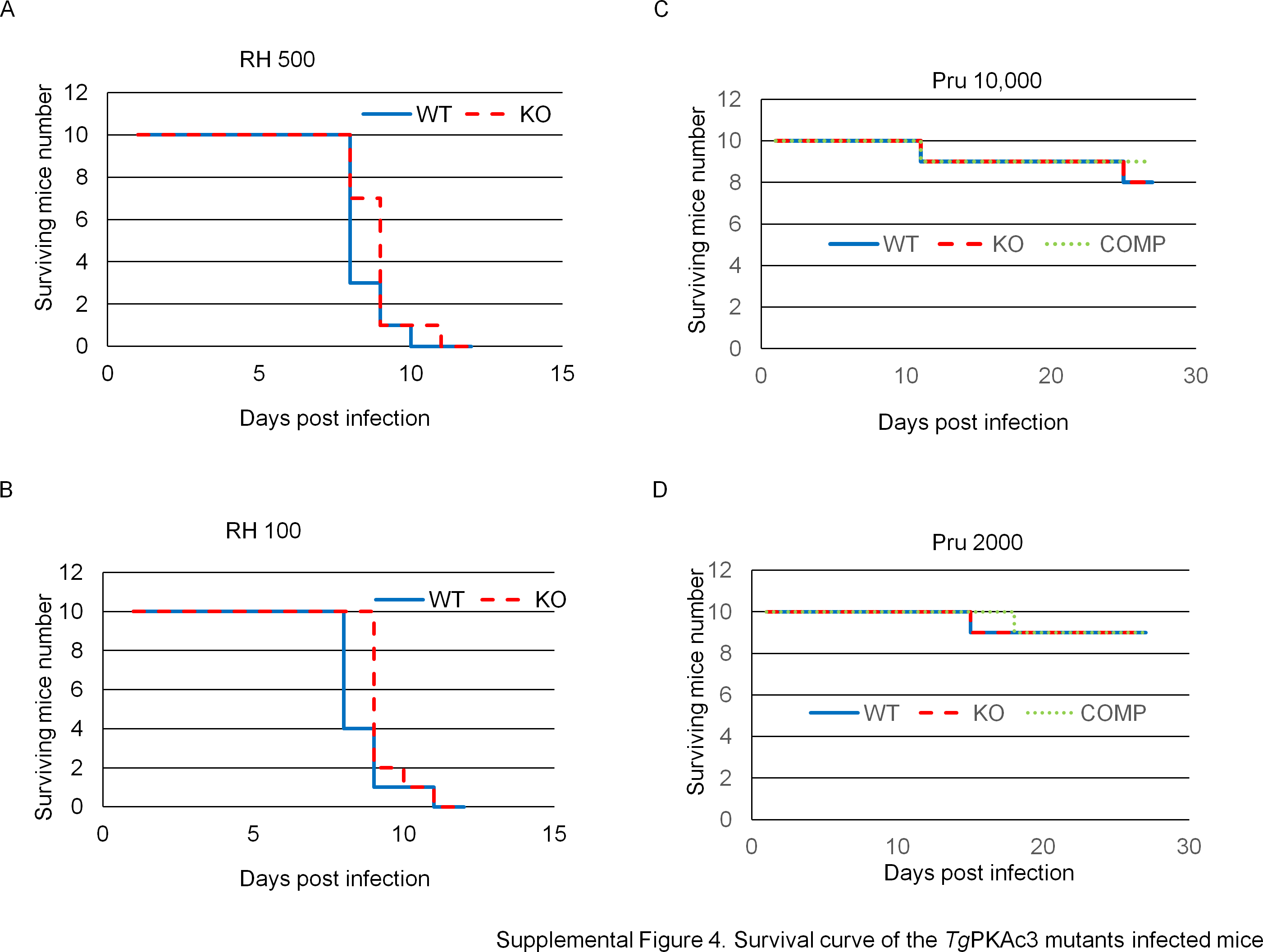

Supplement: Figure S4 — Survival curve of the TgPKAc3 mutant-infected mice. Six- to 8-week-old female C57BL/6J mice were infected with numbers and strains indicated in the figure via the intraperitoneal route (A to D). Ten mice per group were infected and observed daily, and dead mice were recorded until 27 days postinfection. Statistical tests of differences between TgPKAc3 mutants and the parental strain were done with the log rank test. Download [file mbo003162842sf4.tif]
